# Development of experimental chronic kidney disease and vascular calcification alters diurnal variation of phosphate and its hormonal regulators

**DOI:** 10.14814/phy2.14626

**Published:** 2020-11-15

**Authors:** Bruno A. Svajger, Justin L. H. Riddoch, Cynthia M. Pruss, Kimberly J. Laverty, Emilie Ward, Rachel M. Holden, Michael A. Adams

**Affiliations:** ^1^ Department of Biomedical and Molecular Sciences Queen's University Kingston ON Canada; ^2^ Department of Medicine Queen's University Kingston ON Canada

**Keywords:** CKD, diurnal, FGF‐23, phosphate, vascular calcification

## Abstract

The mineral‐bone axis is tightly regulated and dependent on renal function. In chronic kidney disease (CKD) progressive loss of renal capacity disrupts this axis over‐time, with marked changes in circulating calcium, phosphate, PTH, and fibroblast growth factor‐23 (FGF‐23). These changes contribute to the development of cardiovascular disease, like vascular calcification (VC), which worsens morbidity and mortality in CKD. Although the chronic changes in these circulating factors and their relationships are well known, no experimental studies have examined how the progressive development of CKD and VC alter the circadian rhythms of these factors. An adenine‐induced experimental model of CKD in rats was used to establish (i) general circulating trends, (ii) if renal dysfunction affects these observed trends, and (iii) identify potential changes in these trends caused by VC. This study clearly discerned patterns of daily variations in circulating minerals and hormones, finding that both phosphate and PTH follow modelable diurnal variations whereas calcium and FGF‐23 maintain relative stability over 24‐hr. Surprisingly, the development of CKD was not sufficient to disrupt these patterns of diurnal variation and only altered the magnitude of change; however, it was found that the diurnal rhythms of circulating phosphate and daily stability of calcium were only significantly altered in the setting of CKD with established VC.

## Graphical abstract

In this article, we experimentally and progressively generated CKD in rats using an adenine‐based diet. We examined the effect of worsening CKD and the development of vascular calcification on the daily variations in circulating minerals (phosphate, calcium) and mineral‐regulating hormones (PTH, FGF‐23). It was found that CKD did not alter the daily patterns of circulating minerals and hormones, but the occurrence of VC in CKD did.

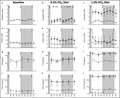

## 1 INTRODUCTION

A biological rhythm is any cyclic change that impacts bodily functions and can involve both endogenous (e.g., temperature) and exogenous controllers (e.g., light, food). Biological rhythms are defined as circadian rhythms if they follow a period of 24 hr (Rosato, (2007). Further characterization based on the interaction between endogenous and exogenous controllers corresponding to an organism's behavior during the day versus the night is known as a diurnal rhythm (Damiola et al., 2000).

Diurnal variations in circulating phosphate levels are well established (Bielesz et al., 2006; Halloran et al., 1985; Ix et al., 2014; Kemp et al., 1992; Markowitz et al., 1981; Portale et al., 1987; Trivedi et al., 2015; Vesely et al., 1996). Circulating phosphate in humans follows a biphasic pattern, with maxima in the middle of both the active (daytime/Light) and resting (nighttime/Dark) phases, with urinary phosphate levels following an inverse of this pattern (Isakova et al., 2012; Ix et al., 2014). This relationship between circulating and urinary phosphate implies that phosphate diurnal variation is significantly dependent on the balance between gut absorption as well as urinary reabsorption and secretion. Specifically, intestinal absorption by Type‐2 sodium‐phosphate cotransporters (Npt‐2b) and renal reabsorption by Npt‐2a transporters (Levi et al., 1994; Miyagawa et al., 2018; Murer et al., 2004; Rizzoli et al., 1977). This diurnal regulation of Npt‐2’s is thought to be independent of either FGF‐23 or PTH regulation since findings by Logue et al. (1990) show that patients with primary hyperparathyroidism and lacking PTH circadian variation still maintain phosphate diurnal variation. Findings suggesting a key role of Napt‐2a/‐2b transporters in the phosphate rhythm may prove contentious as diurnal phosphate variation appears to be preserved in moderate‐to‐severe chronic kidney disease (CKD) (Isakova et al., 2012; Ix et al., 2014; Trivedi et al., 2015). A greater understanding of potential renal independent mechanisms regulating daily oscillations in circulating phosphate is needed.

To date only minor variations in circulating FGF‐23 have been published (Isakova et al., 2008; Miyagawa et al., 2018; Trivedi et al., 2015). With Miyagawa et al. (2018) (Miyagawa et al., 2018) reporting apparent minimal variation in FGF‐23 throughout the day, despite moderate daily heterogeneity in the expression of key FGF‐23 targets such as the Npt‐2 co‐transporters. These findings support the previously established concept that FGF‐23, as a phosphate regulator, is a more long‐term rather than a short‐term regulator of phosphate homeostasis. The findings of Vervloet et al. (2011) also support this concept in showing that repeated administration of a high phosphate diet in healthy adults elevates FGF‐23 slowly, appearing to reset to a higher baseline day‐over‐day.

Calcium and PTH regulation have been better investigated (Blumsohn et al., 1994; Heshmati et al., 1998; Schlemmer & Hassager, 1999). During Dark‐phases (i.e., nighttime for humans), there is a strong dietary impact on the rhythm of calcium, and subsequently PTH (Isakova et al., 2008). During this time period meal timing corresponds to phases of significant increases in the urinary excretion of calcium resulting in a downward trend in circulating calcium (Isakova et al., 2008; Markowitz et al., 1981; Portale et al., 1987), in turn, corresponding to increasing PTH (Isakova et al., 2008; Nielsen et al., 1991). Most often the bathyphase of circulating calcium has been observed during the Dark phase, specifically for humans in the early morning hours (Halloran et al., 1985; Jubiz et al., 1972; Vesely et al., 1996). Correspondingly, PTH maxima occur during this same time period. Blumsohn et al. (1994) identified this peak in PTH as calcium‐dependent, where evening calcium supplementation reverses the PTH peak and only attenuates, but does not reverse, the circadian rhythm of other associated bone resorption markers.

Few clinical studies (Isakova et al., 2008, 2012; Ix et al., 2014; Trivedi et al., 2015; Voogel et al., 2001), and no experimental animal studies have addressed the role of PTH, FGF‐23 or kidney disease with respect to changes in the biological rhythms related to phosphate handling. Given the importance of calcium and phosphate in cellular function and pathogenic processes, it is important to address gaps in knowledge pertaining to the biological rhythms controlling these minerals. Better characterization of daily oscillations would lead to more accurate longitudinal monitoring of CKD patients by selecting sampling points more representative of the 24‐hr average. More importantly, understanding the fluctuations of these minerals and their regulators would improve how and when therapeutic interventions are applied. Be these simple dietary interventions or pharmacologic (e.g., phosphate binders). In the present study, the objective was to determine whether there are substantive changes in the biological rhythms for circulating phosphate, calcium, PTH, and FGF‐23 in a model of adenine‐induced CKD, particularly after an increase in the dietary phosphate and development of vascular calcification.

## 2 MATERIALS AND METHODS

### 2.1 Animal model

All animal procedures were performed in accordance with the guiding principles of the Canadian Council on Animal Care and were approved by the Queen's University Animal Care Committee.

Adult male Sprague Dawley rats (*n* = 20, 14 weeks of age; Hilltop®, Scottdale, PA) were individually housed and maintained on a 12‐hr light/dark cycle. Animals were acclimatized for 1 week prior to study commencement during which they were provided a 0.5% phosphate diet twice daily (2 x 10g; 0.5% phosphate, 1% calcium, 6% protein). Food provision occurred at the same time as lights‐on/lights‐off, 0,730, and 1930. Weekly blood samples (at 1000–1200 hr; 750μL via saphenous venipuncture) were drawn from all animals throughout the entirety of the study. All animals were fed using the twice‐daily delivery protocol (07:30 and 19:30 hr) with water ad libitum throughout the study.

### 2.2 24‐hr sampling, CKD induction, and high phosphate stimuli

At the end the acclimatization period, repeated blood samples were taken (300μL via saphenous venipuncture) every 3 hr for 24 hr (q3h/ 24h) as to characterize baseline diurnal variation in control conditions. After baseline sampling, animals were separated into Control (*n* = 7) and CKD (*n* = 13) groups. CKD was induced by providing a specialized adenine diet BID (2 x 10g; 0.25% adenine, 0.5% phosphate, 1% calcium, 6% protein, *n* = 13) (Shobeiri et al., 2010a).

After 5 weeks of CKD induction, all animals were placed on an adenine‐free low‐normal phosphate diet (2 × 10 g; 0.5% phosphate, 1% calcium, 6% protein). Six days after the diet switch, a q3h/ 24h blood sampling protocol was performed. One day later all animals were placed on a high phosphate diet (2 × 10 g, 1.0% phosphate, 1% calcium, 6% protein) for 6 days after which repetitive blood samples were again collected (q3h/ 24 hr).

On Day 10 of the high phosphate diet, animals were fasted overnight and then anesthetized and received an infusion of radiolabeled phosphate solution (300 μmol in 3ml, Na_2_HPO_4_ containing 100μCi ^33^PO_4_; ~8,000,000 CMP/mL) into the left jugular vein (0.3 ml/min over 10 min). Blood was sampled from the right jugular vein for 30 min. Animals were then euthanized via aortic puncture and exsanguination with tissues excised for analysis.

### 2.3 Serum biochemistry

Serum and plasma samples were spun down after collection and assessed for circulating minerals and hormones. Per kit instructions, plasma was used to measure PTH and iFGF23 levels via ELISA (60‐2500, 60‐6300, Immutopics®, Clemente, CA). Whereas serum was used to measure circulating creatinine with the QuantiChrom Creatinine Assay Kit (DICT‐500; BioAssay Systems, Hayward, CA), while calcium and phosphate were measured using the o‐cresolphthalein complex‐one assay (Sigma‐Aldrich Canada Co., Oakville, ON, Canada) and malachite green methods, respectively (Zelt et al., 2015).

### 2.4 Assessment of tissue mineralization

Tissues (arteries, veins, fat, bone, etc.) were demineralized in hydrochloric acid (50 µl/mg tissue 1.0 N, room temperature) for 1 week. The resultant homogenate was assessed for calcium and phosphate content using the same colorimetric assays used for the analysis of serum calcium and phosphate (McCabe et al., 2013, 2018; Zelt et al., 2015, 2019).

### 2.5 Radioactivity measurement and analysis

Samples from the same pools of tissue homogenate and serum (tissue ‐ 100 μl, serum −200μl) were added to ~5 ml of Ultima Gold AB scintillation cocktail (Perkin Elmer, USA) and analyzed using a Beckman Coulter LS 6500 multi‐purpose scintillation counter. Each sample was measured twice for 1‐min count time. The values were corrected for background and normalized to the total dose of radioactivity given to the rat. Tissue radioactivity for both ^33^phosphate is reported following normalization to the time‐weighted average serum specific activity following cessation of infusion (10 min).

1. Specific Activity (CPM/pmol) = Serum Radioactivity (CPM/uL)/ Serum Phosphate (mM)
2. Tissue Mineral Transport (pmol mineral/mg tissue) = Tissue Radioactivity (CPM/mg tissue)/ Avg Specific Activity (CPM/pmol)

### 2.6 Statistical analysis

Statistical analysis for disease progression, tissue mineral accrual, and patterns of radiolabeled phosphate activity was performed using GraphPad Prism 7.0 (GraphPad Software, La Jolla, CA). Analysis of between‐group differences (e.g., vascular calcium accrual between CKD and Controls) was assessed via unpaired t‐test, whereas multiple group comparisons were performed via Two‐way ANOVA with multiple comparisons and post hoc Tukey correction. A significant difference was defined as *p* < .05. Assessment of variations in the biological rhythms was performed using cosinor analysis as described by Refinetti et al. (2007) using MATLAB®‐R2018a (MathWorks©, Santa Clara, Ca).

## 3 RESULTS

### 3.1 CKD induction and effects of changing dietary phosphate

**Figure 1:**
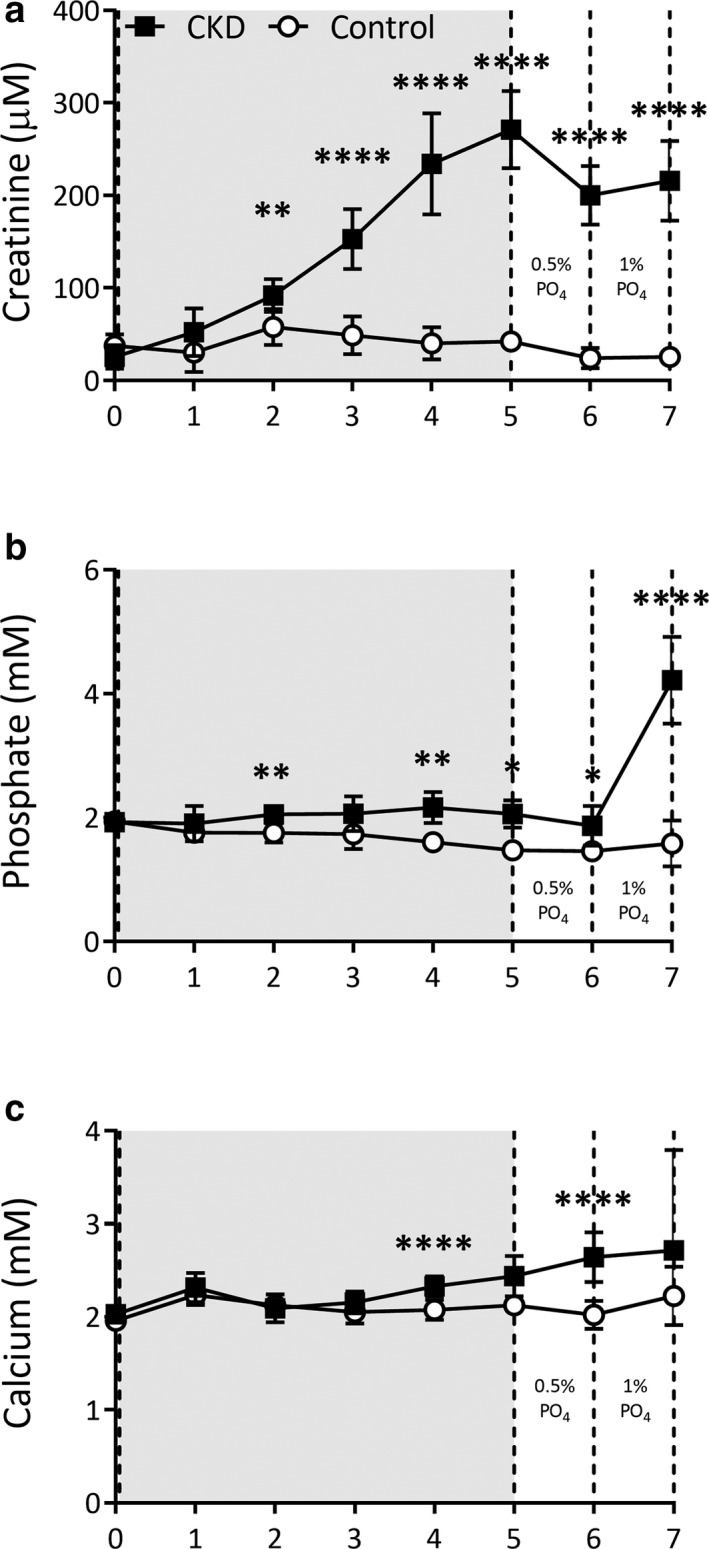
CKD progression as induced by high adenine (0.25%) diet for 5 weeks followed by increasing dietary phosphate challenge week‐over‐week. After 5 weeks on dietary adenine (grey shaded area), rats were switched to a standard 0.5% phosphate diet (same that Controls were on for 5 weeks) for 1 week then all rats were switched to 1.0% phosphate diet for 1 week. CKD rats (black squares, *n* = 13) and Control rats (white circles, *n* = 7). CKD induction led to significant increases in creatinine (a), phosphate (b), and calcium (c). All data are represented as Mean ± *SD*. Groups compared with Unpaired *t* tests. *p < .05, **p < .01, ***p < .001, ****p < .0001 significantly different than Control

**Figure 2:**
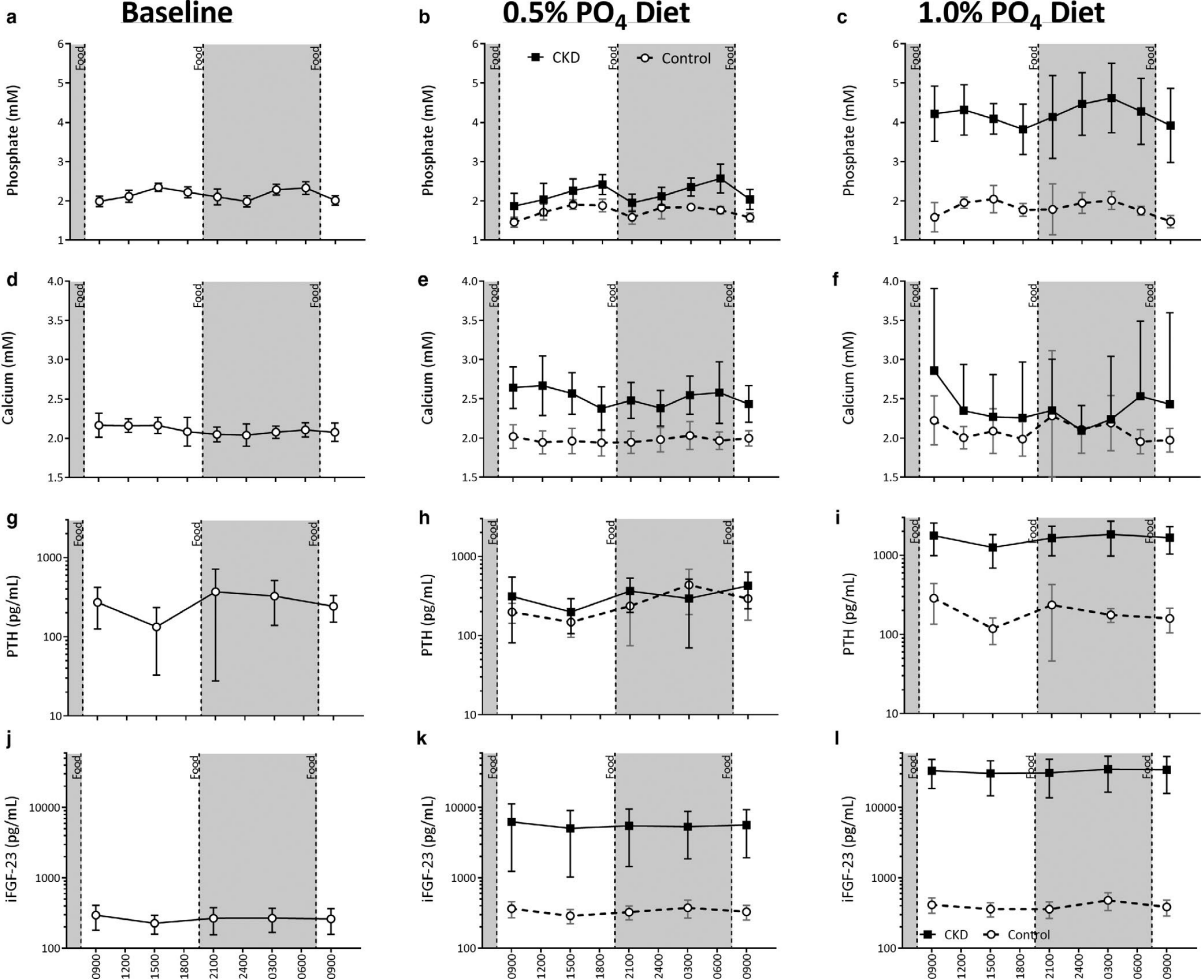
Twenty‐four hour circulating levels of minerals and hormonal regulators in CKD (black squares, *n* = 13) and Control (white circles, *n* = 7) rats. Assessment of phosphate (a‐c), calcium (d‐f), PTH (g‐i), and FGF‐23 (j‐l) at study commencement (a, d, g, j), after CKD induction and 1 week of 0.5% phosphate diet (b, e, h, k) and 1 week of 1.0% phosphate diet (c, f, i, l). Data are represented as Mean ± *SD*. Diurnal variation was assessed by Cosinor analysis

The progressive induction of chronic kidney disease (CKD) using the dietary adenine (0.25%) model was confirmed by the significant elevations to creatinine and phosphate from Week 2 onwards (Figure 1). Attainment of a sufficient decline in renal function, verified when rats were no longer receiving adenine as to avoid confounding by acute kidney injury, was demonstrated at Week 6 by elevated creatinine (Table 1), phosphate (Figure 2b), and FGF‐23 (Figure 2k). PTH was not significantly elevated prior to switching animals onto high dietary phosphate (Table 2).

**Table 1:** Morphological parameters for CKD rats *(fed a 0.25% adenine diet for 5 weeks, n = 13)* and Control rats *(fed a 0.5% phosphate diet for 5 weeks, n = 7),* that were then provided with 1 week of 0.5% phosphate diet and 1 week of 1.0% phosphate diet before sacrifice

	Control (*n* = 7)	CKD (*n* = 13)
Bodyweight (kg)	0.44 ± 0.02	0.37 ± 0.02***
Kidney Mass (g)	2.29 ± 1.00	2.23 ± 0.67
Left Ventricle Mass (g)	0.88 ± 0.03	0.88 ± 0.08
Left Ventricle/ Bodyweight (g/kg)	2.02 ± 0.12	2.37 ± 0.24**
Left Ventricle/ Tibial Length (g/m)	21.12 ± 0.72	21.05 ± 1.76
Phosphate (mM)	1.58 ± 0.14	4.22 ± 0.42***
Calcium (mM)	2.22 ± 0.12	2.72 ± 0.30

Note

Data are represented as Mean ± *SD*. Comparisons made using Unpaired T‐tests. **p < .01, ***p < .001 significantly different than Control.

Abbreviation: CKD, chronic kidney disease.

**Table 2:** Circulating markers of CKD at end of induction, during 0.5% phosphate, and at end of 1.0% phosphate dietary periods in rats with experimental CKD

	End of CKD Induction	End of 0.5% Dietary PO_4_ Phase	End of 1.0% Dietary PO_4_ Phase
Cre	270.51 ± 40.16	200.28 ± 31.86***	215.92 ± 42.92**
Ca	2.39 ± 0.17	2.64 ± 0.27**	2.72 ± 1.08
PO_4_	2.03 ± 0.21	1.87 ± 0.32***	4.22 ± 0.70***

Note

Data are represented as Mean ± *SD*. **p < .01, ***p < .001 significantly different than CKD induction phase.

Abbreviations: CKD, chronic kidney disease; Cre, creatinine (μM); Ca, calcium (mM); PO_4_, phosphate (mM)

**Table 3:** Daily averages of circulating hormones at end of low and high phosphate dietary phases in Control, CKD animals with VC, and CKD animals without VC

	Control (*n* = 7)	CKD Non‐VC (*n* = 6)	CKD VC (*n* = 7)
0.5% PO_4_ Diet – PTH	0.26 ± 0.10	0.29 ± 0.09	0.35 ± 0.23
1.0% PO_4_ Diet– PTH	1.95 ± 0.06	1.45 ± 0.47***†††	1.78 ± 0.71***†††
0.5% PO_4_ Diet – FGF−23	0.33 ± 0.07	2.88 ± 1.14***	7.84 ± 4.19***^‡‡‡^
1.0% PO_4_ Diet – FGF−23	0.40 ± 0.09	18.85 ± 5.71***†††	44.96 ± 12.89***^‡‡‡^ **†††**

Note

Vascular calcification defined as von Kossa stainable aortic mineralization (phosphate >50 nmol/mg tissue, calcium > 80 nmol/mg tissue).

Data are represented as Mean ± *SD*. Groups compared via Multiple Comparisons with Tukey correction.***p < .001 significantly different than Control; ^‡‡‡^ *p* < .05 significantly different than CKD non‐calcified. †††*p* < .001 significantly different than 0.5% dietary phosphate phase.

Abbreviations: CKD, chronic kidney disease; FGF‐23, fibroblast control factor‐23 (ng/mL); Non‐VC, no vascular calcification; PTH, parathyroid hormone (ng/mL); VC, vascular calcification.

Providing high levels of dietary phosphate (1% phosphate diet) led to elevations in circulating phosphate (Figure 2c), PTH (Figure 2i), and FGF‐23 (Figure 2l). In contrast, circulating minerals and hormones were not significantly altered in Control rats given high dietary phosphate.

At sacrifice, animals with CKD had significantly lower bodyweight (*p* < .001) but no change in left ventricular mass, such that the resultant left ventricular to body mass index was significantly increased (*p* < .01, Table 3).

### 3.2 Temporal profile of circulating phosphate

**Figure 3:**
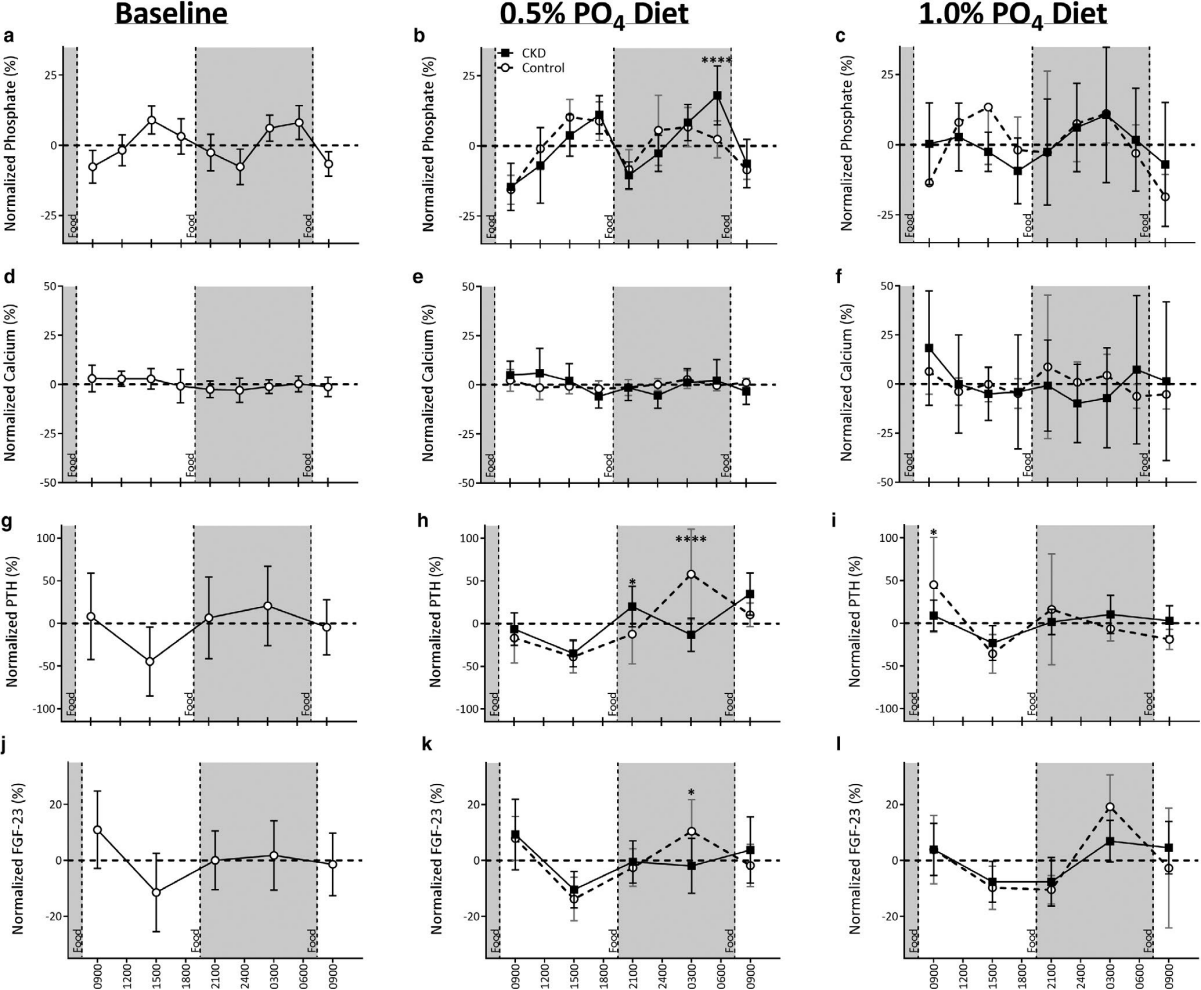
Normalized levels of circulating minerals and hormonal regulators over 24‐hr in CKD (black squares, *n* = 13) and Control (white circles, *n* = 7) rats. Assessment of phosphate (a‐c), calcium (d‐f), PTH (g‐i), and FGF‐23 (j‐l) at study commencement (a, d, g, j), after CKD induction and 1 week of 0.5% phosphate diet (b, e, h, k) and 1 week of 1.0% phosphate diet (c, f, i, l). Data are represented as the percent difference at a given timepoint compared to the animal's circulating daily average using Unpaired T‐tests. *p < .05, ****p < .0001 significantly different from Control. Data are represented as Mean ± *SD*. Diurnal variation was assessed by Cosinor analysis

At baseline, prior to CKD induction (Figures 2a, 3a), the temporal pattern for the circulating levels of phosphate was characterized as having a significant diurnal variation (*p* < .001), modeled with a cosinor profile as described by Refinetti et al. (2007) that was biphasic within each 24‐hr period studied. A similar pattern for the Control group was found at each of the experimental time points parallel to the CKD group after adenine removal (i.e., baseline ≈ 0.5% dietary phosphate ≈ 1% dietary phosphate). That is, a peak was found both during the mid‐to‐late Light cycle (i.e., daytime) and Dark cycle (i.e., nighttime) with the troughs closer to the light‐dark transition phases.

Circulating phosphate was decreased approximately 2 hr after a meal despite the phosphate content and then rose for the remainder of the period prior to the next meal. This pattern was similar in both the Light and Dark phases. Following five‐weeks of CKD induction, with rats no longer consuming adenine but continuing to consume the 0.5% phosphate diet, there was a similar pattern of biphasic peaks compared to Controls. Overall phosphate levels were slightly elevated, particularly during the Dark phase (Figures 2 and 3, *p* < .001). Additionally, the Dark phase phosphate peak was more prominent compared to Controls (Figures 2 and 3). The daily amplitude for circulating phosphate was 0.88 mM (20.18%) in CKD animals and 0.60 mM (17.20%) in Control, with no marked effect of CKD on the normalized amplitude. Switching from the 0.5% phosphate diet to the 1.0% phosphate diet for 1 week in CKD resulted in significant blunting of diurnal variation, particularly with respect to the Light phase phosphate peak. CKD animals displayed a significantly greater amplitude of change (2.0 mM, 24.75%) compared to Control (1.0 mM, 13.18%), and both groups displayed a significantly greater amplitude of change compared to baseline (0.57 mM, 26.2%, *p* < .01 and *p* < .02, respectively).

### 3.3 Patterns in circulating calcium

Circulating calcium showed little 24‐hr variation at baseline or with the induction of CKD (Figures 2d‐f, 3d‐f). No appreciable diurnal variation was observed in either CKD or Control animals during any of the 24‐hr evaluation periods. However, an increased variation in circulating calcium levels was observed in CKD rats after 1 week of 1.0% phosphate diet (*p* < .05). At baseline, an overall difference during Light and Dark phases was present with a calcium elevation during the Light phase and a slight decrease during Dark periods (*p* < .01, Table 4). The relative change in the circulating level of calcium was unaffected by CKD although 1.0% phosphate diet (Table 5) increased levels compared to baseline and 0.5% phosphate (*p* < .001). There was no significant impact on calcium in Control animals when put on the 1.0% phosphate diet.

**Table 4:** Circulating averages for minerals and hormonal regulators in animals (*n* = 20) at study commencement

	Daily average	Amplitude (%)	Light phase average	Dark phase average
PO_4_	2.16 ± 0.08	26.15 ± 6.01	2.14 ± 0.07	2.18 ± 0.12
Ca	2.10 ± 0.04	18.15 ± 7.24	2.13 ± 0.05	2.07 ± 0.06^ψψ^
PTH	0.26 ± 0.10	135.85 ± 60.85	0.22 ± 0.07	0.35 ± 0.24^ψψ^
FGF−23	0.26 ± 0.10	36.21 ± 17.76	0.26 ± 0.09	0.26 ± 0.11

Note

Data are represented as Mean ± *SD*. Light and Dark phases compared with Unpaired T‐test. ^ψψ^ *p* < 0.01 significantly different than Light phase.

Abbreviations: Ca, calcium (mM); FGF‐23, fibroblast growth factor‐23 (ng/mL); PO_4_, phosphate (mM); PTH, parathyroid hormone (ng/mL).

**Table 5:** Circulating averages for minerals and hormonal regulators in CKD rats *(5 weeks 0.25% adenine diet prior to switch)* and Control rats after 1 week of 0.5% phosphate diet followed by 1 week of 1.0% phosphate diet

	CKD (*n* = 13)				Control (*n* = 7)			
	Daily Average	Max‐Min Amplitude (%)	Light Phase	Dark Phase	Daily Average	Max‐Min Amplitude (%)	Light Phase	Dark Phase
0.5% Diet – PO_4_	2.18 ± 0.23	40.4 ± 11.87	2.12 ± 0.25	2.25 ± 0.22	1.73 ± 0.10	34.40 ± 8.59	1.71 ± 0.11	1.76 ± 0.11
1.0% Diet – PO_4_	4.21 ± 0.42	49.5 ± 28.89	4.08 ± 0.42	4.38 ± 0.49^ψ^	1.81 ± 0.14	53.41 ± 19.77	1.76 ± 0.14	1.87 ± 0.21
0.5% Diet – Ca	2.52 ± 0.19	27.2 ± 8.10***	2.54 ± 0.19	2.50 ± 0.20	1.98 ± 0.12	13.57 ± 4.77	1.97 ± 0.12	1.98 ± 0.14
1.0% Diet – Ca	2.38 ± 0.32	84.9 ± 39.30***	2.44 ± 0.42	2.31 ± 0.29	2.09 ± 0.17	37.39 ± 32.24	2.05 ± 0.18	2.13 ± 0.25
0.5% Diet – PTH	0.32 ± 0.17	83.3 ± 27.26	0.31 ± 0.16*	0.32 ± 0.19	0.26 ± 0.10	109.86 ± 54.08	0.22 ± 0.08	0.34 ± 0.17
1.0% Diet – PTH	1.63 ± 0.61	56.0 ± 21.40*	1.56 ± 0.57	1.73 ± 0.70	0.19 ± 0.05	114.17 ± 69.25	0.19 ± 0.07	0.21 ± 0.10
0.5% Diet – FGF−23	5.55 ± 4.0	31.0 ± 12.33	5.65 ± 4.18	5.40 ± 3.73	0.33 ± 0.07	29.81 ± 15.39	0.33 ± 0.07	0.35 ± 0.08
1.0% Diet – FGF−23	32.90 ± 16.73	25.44 ± 8.90***	32.78 ± 16.05	33.11 ± 17.88	0.40 ± 0.09	44.84 ± 9.72	0.38 ± 0.09	0.42 ± 0.11

Note

Data are represented as Mean ± *SD*. Diurnal variation was assessed by Cosinor analysis. Phases compared using Unpaired T‐test. *p < .05, ***p < .001 significantly different than Control; ^ψ^ *p* < 0.05 significantly different than Light phase.

Abbreviations: Ca, calcium (mM); CKD, chronic kidney disease; FGF‐23, fibroblast growth factor‐23 (ng/mL); PO_4_, phosphate (mM); PTH, parathyroid hormone (ng/mL).

### 3.4 Patterns in circulating PTH

At baseline, PTH levels presented with diurnal variation (*p* < .05, Figures 2g, 3g). In the Light phase, PTH levels fell relative to the daily average while levels rose during the Dark phase, resulting in significant differences between the two phases (Figures 2 and 3, Table 4). The largest decline in circulating PTH occurred mid‐afternoon in the Light phase of all three experimental periods. A diurnal pattern of variation was still present in CKD animals after 1 week on the 0.5% phosphate diet (*p* < .001, Figures 2h, 3h). Except for a transient nighttime dip in PTH levels in these CKD rats, there was a similar overall pattern compared to baseline, with the Light phase corresponding to a period of relative decline and the Dark phase to overall increase (*p* < .001, Table 5). Both CKD and Control groups displayed a change in circulating PTH diurnal variation after 1 week of consuming 1.0% phosphate diet (*p* < .05 and *p* = .001, respectively; Figures 2h, 3h). Significant differences between Light and Dark phases were still present in CKD animals (*p* < .05, Table 5), but not in Control rats. As with earlier experimental periods, the greatest decreases were toward the latter portion of the Light phase.

### 3.5 Patterns in circulating FGF‐23

At baseline, FGF‐23 presents with a marked diurnal variation (*p* < .05, Figures 2j, 3j). The amplitude of daily variation to FGF‐23 was smaller than in PTH. CKD progression and consumption of the 0.5% phosphate diet resulted in a significant pattern of diurnal variation (*p* < .001) although the amplitude of FGF‐23 was significantly reduced in the Dark phase of CKD animals compared to Controls (Figures 2k, 3k, Table 5). Increasing dietary phosphate content to 1.0% attenuated the previous diurnal variation for FGF‐23 in the CKD animals, while a significant diurnal variation was maintained in Control animals (Figures 2l, 3l). While on the 1.0% diet both groups demonstrated a slight prolongation of the trough that spanned from the Light phase into the early Dark phase.

### 3.6 Vascular mineral content in CKD animals at sacrifice and ad hoc stratification by VC

**Figure 4:**
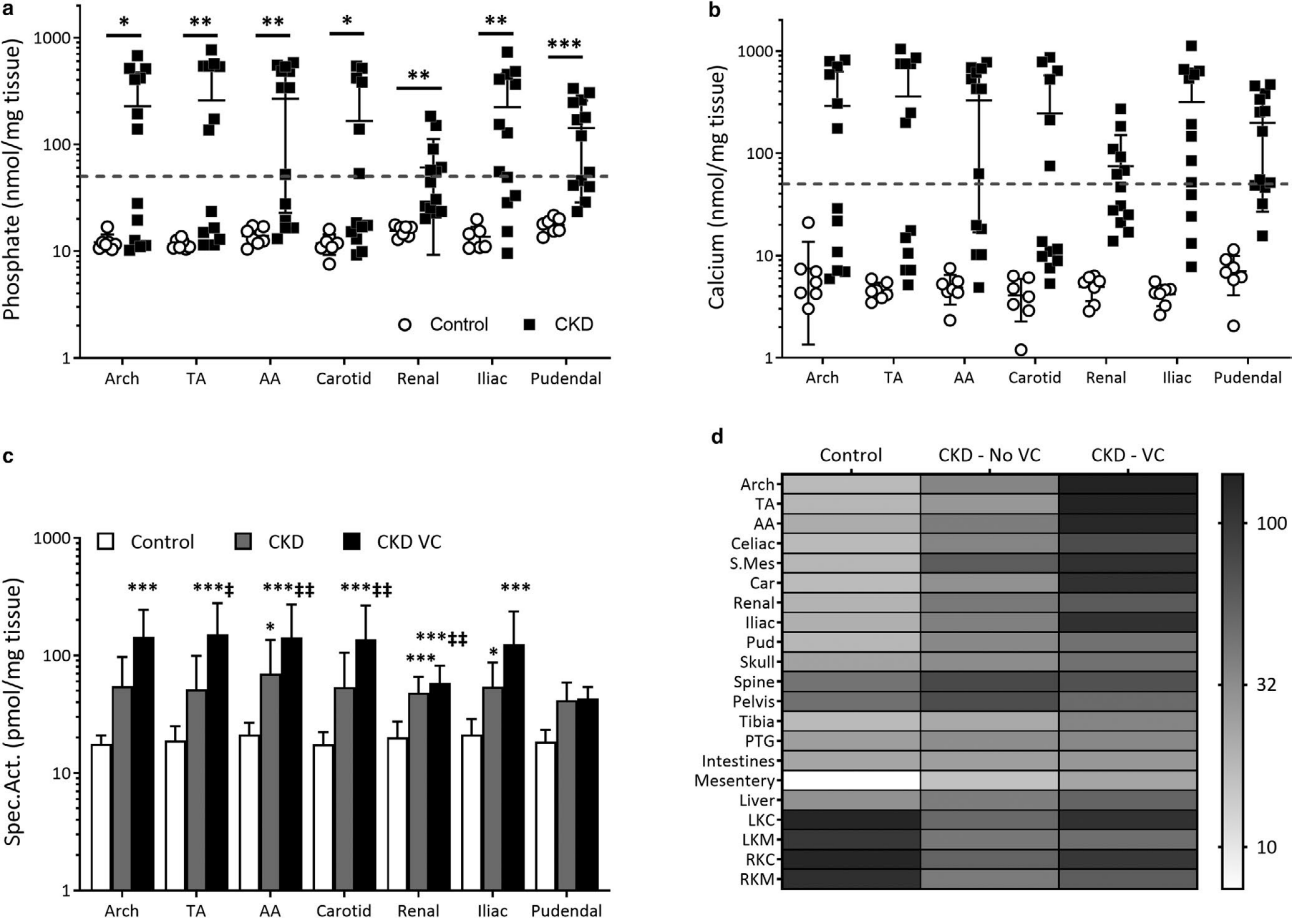
Phosphate accrual dynamics in Control (*n* = 7) and CKD rats (*n* = 13) and the effect of vascular calcification. Increased vascular mineral accrual represented by phosphate (a) and radiolabeled phosphate accrual (b) across vascular beds. Heat map (c) representing radioisotope accumulation generated through rank‐ordering and averaging sites of greatest (dark shading) versus least accrual (light shading). VC defined as von Kossa stainable aortic calcification (aortic phosphate >50 nmol/mg tissue). Data are compared using Unpaired T‐tests (a, b) and Multiple‐Comparisons with Tukey correction (c). *p < .05, **p < .01, ***p < .001 significantly greater compared to Control. ‡*p* < .05, ‡‡*p* < .01 significantly different than CKD non‐calcified. Data are represented as Mean ± *SD*

Vascular content of phosphate (Figure 4a) and calcium (Figure 4b) significantly elevated across all vascular beds in CKD animals compared to Control (*p* < .001). 62% of CKD animals experienced vascular calcification (VC) based on aortic‐vascular mineralization levels that correspond to levels identifiable with von Kossa staining (phosphate ≥50 nmol/mg, calcium ≥80 nmol/mg). An *ad hoc* analysis was conducted using these criteria to examine the effects of VC on circulating mineral‐hormonal characteristics in CKD animals. CKD animals were categorized as calcified (VC, *n* = 7) or non‐calcified (No VC, *n* = 6). No differences in kidney function, physical characteristics or circulating creatinine, phosphate, calcium or PTH were observed (Table 6). Circulating FGF‐23 was significantly higher in the VC group compared to Non‐VC (*p* < .001).This difference in circulating FGF‐23 was present both prior and after switching to the 1.0% phosphate diet (*p* < .001, Table 2).

**Table 6:** Comparison of Calcified versus Non‐Calcified CKD rats at the end of the study

	VC (*n* = 7)	No VC (*n* = 6)
Bodyweight (kg)	0.37 ± 0.01	0.38 ± 0.01
Kidney Mass (g)	2.23 ± 0.26	2.24 ± 0.30
Left Ventricle Mass (g)	0.89 ± 0.03	0.87 ± 0.04
Left Ventricle/ Bodyweight (g/kg)	2.44 ± 0.10	2.28 ± 0.08
Left Ventricle/ Tibial Length (g/m)	21.12 ± 0.70	20.97 ± 0.75
Cre	209.10 ± 49.13	223.88 ± 37.19
PO_4_	4.37 ± 0.88	4.04 ± 0.43
Ca	3.05 ± 1.33	2.32 ± 0.57
PTH	1.78 ± 0.27	1.45 ± 0.19
FGF−23	45.00 ± 4.87^‡‡‡^	18.85 ± 2.33

Note

Vascular calcification defined as von Kossa stainable aortic mineralization (phosphate > 50 nmol/mg tissue, calcium > 80 nmol/mg tissue).

Data are represented as Mean ± *SD*. Groups compared using Unpaired T‐test. ^‡‡‡^ *p* < .001 significantly different than CKD non‐calcified.

Abbreviations: Ca, calcium (mM); CKD, chronic kidney disease; Cre, creatinine (μM), PO_4_, phosphate (mM); FGF‐23, fibroblast growth factor‐23 (ng/mL); PTH, parathyroid hormone (ng/mL).

### 3.7 Acute regulation of infused radiolabeled phosphate (^33^PO_4_)

Previous investigations demonstrated that acute handling of minerals is significantly altered in CKD and differentially impacted by the presence of VC (Zelt et al., 2019). In this study, radiolabeled phosphate (^33^PO_4_) was infused to assess the acute handling of phosphate between groups. Deposition of phosphate was determined after adjusting for the specific activity of circulating radioactive phosphate. Overall vascular accrual of ^33^PO_4_ was significantly elevated in CKD compared to Control (*p* < .01, Figure 4c). The heat map (Figure 4c) of collected tissues revealed that in CKD the major change compared to baseline for acute ^33^PO_4_ deposition was the greater accrual in vascular tissue, even though kidneys, bone, intestine, and liver substantially accrue phosphate in Controls. Further comparison of acute deposition between calcified and non‐calcified CKD animals (Figure 4b) revealed that there was further enhancement of vascular phosphate accrual in calcified animals.

### 3.8 Effect of vascular calcification on diurnal variation

**Figure 5:**
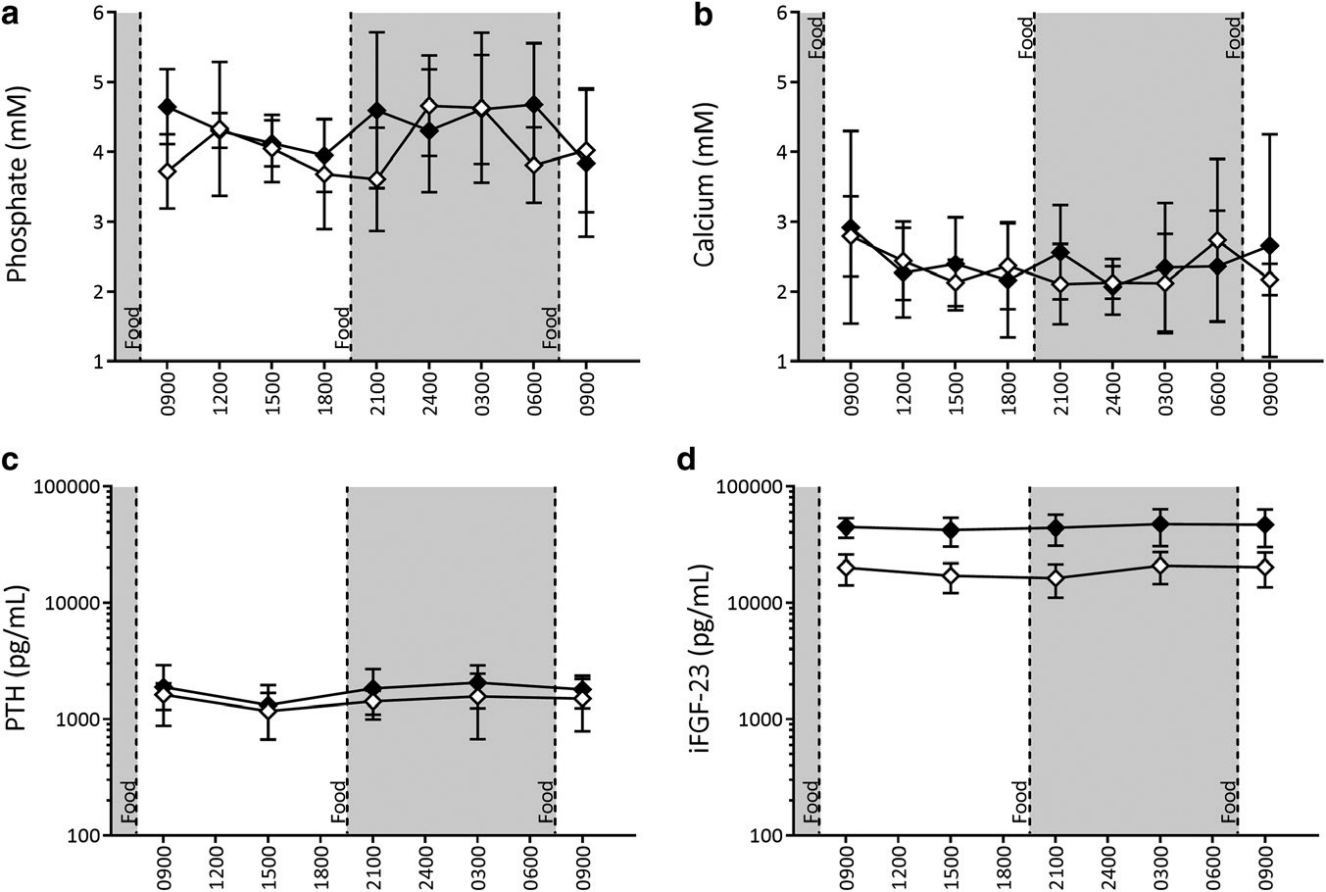
Effect of vascular calcification on 24‐hr levels of circulating minerals and hormonal regulators in rats consuming 1% dietary phosphate. Comparison of CKD animals with vascular calcification (filled diamonds, *n* = 7) versus those without (empty diamonds, *n* = 6) of circulating markers of CKD. Data are represented as Mean ± *SD*

**Figure 6:**
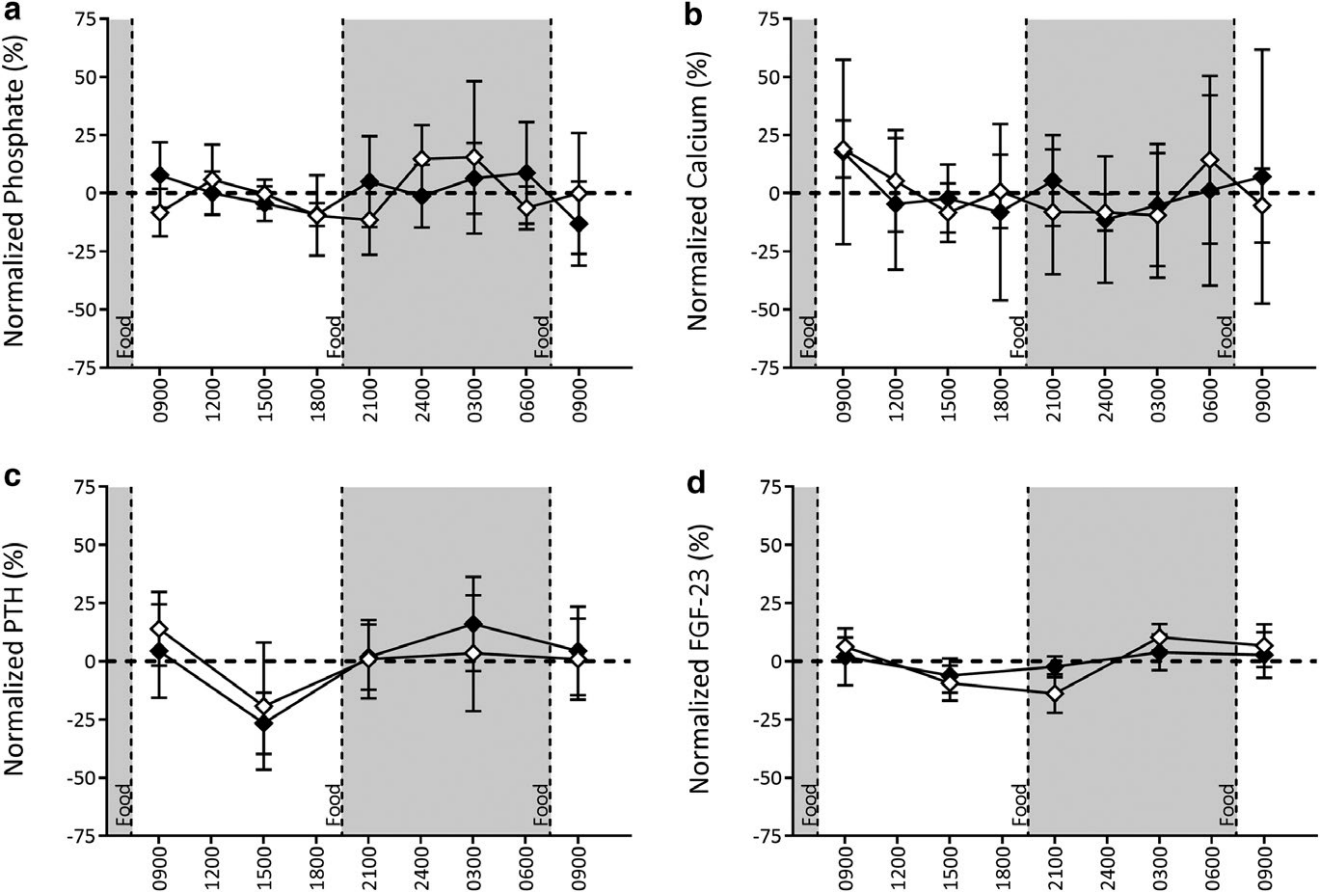
Effect of vascular calcification on 24‐hr levels of circulating minerals and hormonal regulators in rats consuming 1% dietary phosphate. Comparison of CKD animals with vascular calcification (filled diamonds, *n* = 7) versus those without (empty diamonds, *n* = 6) of normalized circulating markers of CKD. Data are represented as the percent difference at a given timepoint compared to the animal's circulating average. Data are represented as Mean ± *SD*. Diurnal variation was assessed by Cosinor analysis

Previous studies indicate VC associated with CKD normally requires a high dietary phosphate stimulus (El‐Abbadi et al., 2009; Hortells et al., 2017). Stratifying CKD animals based on the endpoint presence or absence of VC showed that there was a diurnal variation present for phosphate in non‐calcified rats (*p* < .01, Figures 5a, 6a) whereas no variation was found in those with VC. Specifically, in non‐calcified animals the previously reported biphasic pattern in circulating phosphate with an initial peak present in the early afternoon and the greater peak mid‐evening. Neither group demonstrated significant diurnal variation in PTH or FGF‐23 (Figures 5c‐d, 6c‐d). A phase‐dependent variation in PTH and FGF‐23 was observed in both groups (*p* < .01 and *p* < .001, respectively).

## 4 DISCUSSION

This study sought to characterize how daily variations in circulating minerals (phosphate, calcium) and hormonal regulators (PTH, FGF‐23) may be affected by CKD and the development of vascular calcification (VC). To our knowledge, this is the first study to compare and contrast the effect of experimental CKD with and without VC on the daily variation of these circulating factors. The major findings were that (i) renal impairment did not markedly alter normal daily variations in circulating minerals or hormonal regulators in low‐normal dietary phosphate conditions, (ii) that the generation of severe hyperphosphatemia in CKD was associated with an attenuated diurnal variation in phosphate and PTH, (iii) the development of VC in CKD was predicted by an early increase in the circulating level of FGF‐23, but not PTH or hyperphosphatemia, and (iv) the presence of VC associated with an altered diurnal variation in circulating phosphate.

Consistent with previous studies the use of the dietary adenine‐induction model of CKD resulted in the progressive development of renal dysfunction with corresponding elevations to circulating creatinine, phosphate, calcium, and FGF‐23 (McCabe et al., 2013, 2018; Shobeiri et al., 2010b; Zelt et al., 2015, 2019). A high phosphate diet was required to induce severe hyperphosphatemia as well as marked elevation of PTH and FGF‐23 in the CKD rats. An incidental finding in this study was the early threefold elevation to FGF‐23 in the that went on to develop VC after 5 weeks of CKD progression, prior to the provision of a VC‐inducing high phosphate diet. Although the specific role of FGF‐23 in the development of cardiovascular disease remains unresolved, this early elevation pre‐hyperphosphatemic stimulus may be an index of susceptibility for vessels to calcify or identify vessels that are in the early phases of VC with transdifferentiating vascular smooth muscle cells (VSMCs) that are producing FGF‐23. As such, FGF‐23 may act as an early marker of VC prior to the development of more severe calcification. Further evaluation is necessary to better understand the timing of these changes and impact on vascular physiology.

In agreement with clinical studies, healthy control rats exhibited diurnal variations in both phosphate and PTH that were similar to previously established trends (i.e., biphasic peaks in phosphate, Dark phase peak in PTH); additionally, although of lesser amplitude, FGF‐23 also presented with a diurnal rhythm. No clear diurnal variation could be detected for calcium under control conditions, although, a decreasing trend similar to previously established patterns was observed (Blumsohn et al., 1994; Calvo et al., 1991; Eastell et al., 1992; Markowitz et al., 1981; Roelfsema et al., 1980). These findings, particularly the PTH rise corresponding to lower calcium, were anticipated based on previous reports (Blumsohn et al., 1994). In the current study, animals were provided with the same amount of food at the onset of both the Light and Dark phases to mitigate the potential effect of fasting periods on daily mineral regulation. Rats are nocturnal and as such, it was unexpected that the pattern was not inverted between Light and Dark compared to humans (i.e., period of calcium decrease and PTH increase during daylight, aka lights‐on). Based on this observation, clinically, the relative importance in roles of light‐dark cycles versus the timing of food consumption needs to be better resolved. One complication of the approach used herein relates to the possible impact of blood sampling causing stress to the animals, even though stress was not previously shown to affect diurnal variations of calcium, PTH or bone resorption (Heshmati et al., 1998).

Five weeks of CKD induction with adenine plus an additional week on the adenine‐free 0.5% phosphate diet yielded no alterations to the observed diurnal variations at baseline. These results are in line with clinical examinations in CKD patients (Isakova et al., 2008; Ix et al., 2014; Trivedi et al., 2015). Of note, is a potential deficiency within the vitamin D metabolome given the observed marked increase to circulating FGF‐23 at this timepoint despite only moderate increases in phosphate and creatinine. Appropriate indicators of vitamin D receptor activation will need to be developed since circulating levels of calcitriol alone are not sufficient (Kaufmann et al., 2018). No appreciable changes in diurnal variation compared to baseline were observed in phosphate or calcium until high dietary phosphate was given and/or VC occurred. Although these results, at least in part, contradict the findings of Miyagawa et al. (2018) the basis of the discordance is not self‐evident unless the regulation of Npt‐2 in the intestines and kidneys play a larger role in the diurnal variation of these minerals and hormones than previously thought (Miyagawa et al., 2018). A limitation in this study was an inability to evaluate intestinal or renal Npt‐2 expression and is an area in need of further research, although a significant impact on intestinal phosphate absorption was not expected with CKD (Marks et al., 2007).

Dietary phosphate was also increased in Control animals to try to identify potential effects of phosphate exposure on normal daily mineral homeostatic patterns. In healthy Controls, the increase to dietary phosphate did not alter the patterns of phosphate or FGF‐23, but did result in a left‐shift of the PTH maxima by 3 hr and a blunting of the overall variation profile. Unlike at baseline, a diurnal variation was observed in calcium with a peak occurring at the start of the Dark phase. This result was unexpected as calcium generally decreases with a phosphate stimulus in addition to decreasing postprandially (Isakova et al., 2008). The increased calcium levels may explain the observed decrease in relative circulating PTH.

With the CKD animals, the 1.0% phosphate diet was used to induce a severe hyperphosphatemic state that altered the daily phosphate rhythm. CKD progression and addition of high dietary phosphate did yield significant increases to circulating FGF‐23, yet did not significantly alter the diurnal variation of FGF‐23. It was hypothesized that the large change was a consequence of changes in several other factors (e.g., vitamin D deficiency) that would have generated a more noticeable alteration in daily variability, but it may be that the magnitude of the change represented a maximal signal for FGF‐23 production and release. Overall in terms of the mineral‐bone axis, it appears that FGF‐23 has a role that is not affected by acute daily changes, unlike PTH (Ledger et al., 1995; Trivedi et al., 2015; Vervloet et al., 2011). With respect to calcium, the more prominent decrease in this mineral is likely due to changes in the acute mineral handling reservoirs where instead of calcium and phosphate interacting with bone they now exchange with the vasculature (Zelt et al., 2019).

Inducing hyperphosphatemia in the CKD animals served two roles: first, it enabled the evaluation of an increased phosphate load across 24 hr and, second, it induced VC to enable the assessment of the impact of VC on biological rhythms. To verify that VC significantly altered acute mineral handling in this study we infused radiolabeled phosphate, collected multiple blood samples over 30‐min, then sacrificed animals and harvested tissues to analyze locations of accrual. The 30‐min timeframe was selected as this is a renal‐independent window for the clearance of phosphate from the circulation (Zelt et al., 2019). Tissue analysis revealed that in CKD animals with VC a preferential deposition for phosphate into the arteries occurs, contrasting the preference for bone in non‐calcified animals, and the kidneys and bone for Controls. The preferential phosphate deposition in arteries in CKD is likely the result of vascular smooth muscle cells transdifferentiating to osteoblastic phenotypes (Jono et al., 2000; Reynolds et al., 2004), and appears to be further increased when vessels calcify and become more susceptible to mineral deposition (Zelt et al., 2019).

Given these altered mineral dynamics and the variable presence of VC, the daily variations were re‐examined by stratifying based on the presence or absence of VC. In CKD with VC, there was less of a pattern of daily phosphate and FGF‐23 variation but more variation with PTH. For CKD animals without VC a return to regular variation in phosphate was observed, indicating that the condition and progression of VC likely alter the normal mechanisms for regulating phosphate diurnal variation. This finding is likely explained by the calcified vasculature in CKD becoming a preferred acute depot for mineral exchange, compared to bone in healthier conditions.

A significant repercussion of mineral dysregulation in CKD is the occurrence of renal osteodystrophy. This results in weakened bone and overall frailty that can contribute to fractures (Goodman et al., 1994; Martin & Gonzalez, 2007). Further, significant elevations in circulating phosphate and FGF‐23 in CKD have been shown to associate with increased morbidity and mortality, particularly from cardiovascular disease (Ix et al., 2012; Kestenbaum et al., 2005; London et al., 2007; Mirza et al., 2009). Thus, some therapeutic strategies in CKD aim to mitigate hyperphosphatemia, thereby attenuating the progression toward cardiovascular sequelae and preserving bone health (Isakova et al., 2013; Ketteler et al., 2017). By better understanding how these minerals and their hormonal regulators normally vary throughout the day as well as how the regulation is altered by CKD, more targeted therapeutic approaches can be developed. The present findings provide evidence that there should be specific times selected for sample collection given the observed patterns, particularly if the objective is a long‐term versus short‐term indicator. As the first investigation in experimental CKD to assess the daily variations in these factors, this study was able to demonstrate that: (i) renal phosphate handling does not appear to be the dominant component in these daily variations, (ii) severe hyperphosphatemia in CKD is associated with altered phosphate, calcium, and PTH variability, and (iii) VC significantly decreases the daily oscillations of circulating phosphate and FGF‐23, while increasing the variation in PTH. Taken together, the findings provide a framework to examine the short‐term and long‐term time course of changes in the minerals and hormones, highlighting that the patterns of change likely have different time constants.

## CONFLICT OF INTEREST

Michael A. Adams is currently in receipt of an educational grant from OPKO Health Inc. Renal Division for a different project.

## AUTHOR CONTRIBUTIONS

BAS conducted primary study design, data collection, generation of pertinent figures and tables and was the primary author for this manuscript. CMP and EW provided technical assistance for serum/plasma and tissue analysis. KL provided technical assistance with animal handling, daily checks, and tissue collection. JLHR assisted with data analysis. RMH and MAA assisted with study design, analysis, and editing of the manuscript.
